# Portable Negative Pressure Wound Dressing in Oncoplastic Conservative Surgery for Breast Cancer: A Valid Ally

**DOI:** 10.3390/medicina59101703

**Published:** 2023-09-23

**Authors:** Donato Casella, Daniele Fusario, Anna Lisa Pesce, Marco Marcasciano, Federico Lo Torto, Gianluigi Luridiana, Alessandro De Luca, Roberto Cuomo, Diego Ribuffo

**Affiliations:** 1 Department of Medicine, Surgery and Neurosciences, University of Siena, 53100 Siena, Italy; donato.casella@unisi.it (D.C.); annalisa.pesce@student.unisi.it (A.L.P.); roberto.cuomo@unisi.it (R.C.); 2 Unit of Plastic and Reconstructive Surgery, University of Catanzaro “Magna Graecia”, 88100 Catanzaro, Italy; dott.marcomarcasciano@gmail.com; 3 Department of Plastic Reconstructive and Aesthetic Surgery, Sapienza Università di Roma, 00185 Rome, Italy; federico.lotorto@uniroma1.it (F.L.T.); diego.ribuffo@uniroma1.it (D.R.); 4 Unit of Oncologic and Breast Surgery, A.R.N.A.S Brotzu, Businco Oncologic Hospital, 09047 Cagliari, Italy; gianluigi.luridiana@aob.it; 5 Department of Surgery, Sapienza Università di Roma, 00185 Rome, Italy; dr.aless.deluca@gmail.com

**Keywords:** wound healing, oncoplastic, breast cancer, negative pressure

## Abstract

*Background and Objectives*: The use of oncoplastic techniques has spread widely in the last decade, with an expansion of the indications and demonstration of excellent oncological safety profiles. A potential downside may be the increased complication rates, which could influence the timing of adjuvant therapy. To date, there is increasing evidence that negative pressure therapy on closed wounds can reduce complication rates after surgery. From this perspective, we tested the use of portable negative pressure wound dressings (NPWDs) in oncoplastic surgery to minimize early post-operative admissions to the outpatient clinic and prevent surgical complications. *Materials and Methods:* An observational prospective cohort study was conducted on a population of patients who underwent quadrantectomy and wise-pattern reduction mammoplasty for breast cancer. The primary objective of the study is represented by the evaluation of the impact of NPWD on post-operative outcomes in an oncoplastic surgery setting. Patients enrolled between January 2021 and January 2023 were divided into two groups, the conventional dressing (CD) group and the NPWD group, by a simple randomization list. *Results*: A total of 100 patients were enrolled, with 52 in the CD group and 48 in the NPWD group. The use of NPWD significantly reduced the wound dehiscence rate (2.0% vs. 7.7% *p* = 0.002) and the number of one-month postoperative admissions to our clinic (3.8 ± 1.1 vs. 5.7 ± 1.3 *p* = 0.0009). Although not significant, it is possible to note a trend of reduction of clinically relevant postoperative total complications in patients treated with NPWDs. *Conclusions*: NPWDs may represent a useful tool in the post-surgical management of complex oncoplastic procedures, ensuring less wound dehiscence. Furthermore, the use of these dressings led to a significant reduction in admissions to the clinic, promoting a lower use of resources by hospitals and effective prevention of possible complications.

## 1. Introduction

Immediate breast reconstruction after breast-conserving surgery has been a significant innovation in the treatment of breast cancer: its success is based on the combination of complete excision of tumors with adequate and oncologically safe margins with the attempt to preserve the natural shape and appearance of the breast. The implementation of screening and neoadjuvant chemotherapy has extended the indications for breast-conserving surgery.

Despite the proven oncological safety, oncoplastic breast surgery was introduced to overcome the obstacle of unsatisfactory aesthetic results. Oncoplastic breast-conserving surgery aims to increase the satisfaction and psychosocial well-being of patients with breast cancer by guaranteeing them the best breast reconstruction in the context ofdemolitionsurgery [1,2]. The use of oncoplastic techniques has spread widely in the last decade, with an expansion of the indications [3,4,5] and demonstration of excellent oncological safety profiles [6,7], also guaranteeing a greater resection volume achieved compared to conventional tumorectomy [8] and an increase in patient satisfaction and quality of life [9,10,11,12,13].

There are currently several classification systems for oncoplasty techniques, such as Hoffmann–Wallwiener [14], Clough [15], and ASBrS [16], essentially based on the amount of excised volume and the consequent mobilization.

A potential downside of oncoplastic surgery may be the increased complication rates [9,12,17], reported in 13–31% of patients [18,19,20]. In a review of 11 prospective studies, the complication rate was approximately 20% (divided into early (<2 months), including abscess, seroma, dehiscence, delayed wound healing, hematoma, partial skin necrosis, infection, and late (>2 months), including breast fibrosis, fat necrosis, hypertrophic scarring, and radiation burn) [21]. This high rate of complications, which could influence the timing of adjuvant therapy, means that great attention is paid to identifying techniques and systems capable of minimizing complications in a preventive manner.

Although oncoplastic surgery, due to the complexity of the procedures implemented, may result in higher rates of post-surgical complications and consequently increased patient admissions to post-surgical clinics, it allows for more conservative procedures with improved functional and cosmetic results [2,6,22,23].

Negative pressure dressing found its first application in the 19th century for wound care, initially in thoracic surgery, to prevent collapsed lungs [24]. The form most in use today comes from the 1987 development work of Morykvas et al., based on a polyurethane sponge and a machine that applies negative pressure to it, the results of which were published in 1997 [25]. From these systems, portable systems were developed that reduce the size of the vacuum machine and combine dressing and reservoir functions. Their use was applied early in breast surgery. There is increasing evidence that negative pressure therapy on closed wounds can reduce complication rates after surgery by reducing lateral tension [26], thereby lowering the surgical site infection rate and strengthening the wound closure. Increasing tissue perfusion and oxygenation aids in healing, angiogenesis, collagen production, and granulation tissue formation. It has also been shown to improve lymphatic drainage, reducing the development of hematomas, seromas, and tissue edema [27,28,29,30,31].

From this perspective, we tested the use of portable negative pressure wound dressings (NPWDs) in oncoplastic surgery. We expect that NPWDs, through vacuum therapy induced by a disposable device that applies −80 mmHg of pressure through a self-sealing dressing, which also acts as a reservoir, ensure a lower rate of early post-operative admission to outpatient clinics and prevent surgical complications [32,33,34]. To the best of our knowledge, this study is one of few that have evaluated the use of NPWDs as a device to reduce postoperative outpatient visits beyond evaluating its impact on wound healing.

## 2. Materials and Methods

**Figure 1:**
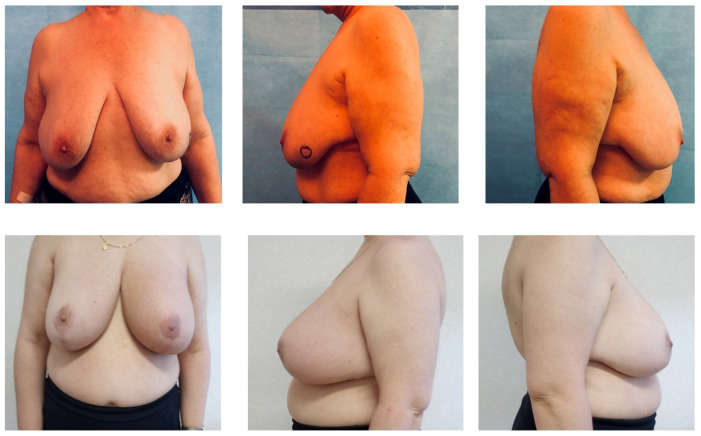
Preoperative and one-month follow-up breasts.

Oncoplastic surgery was taken into consideration whenever an excision greater than 20% of the volume of the breast parenchyma was expected (Table 1). When indicated, simultaneous contralateral symmetrization was proposed (Figure 1).

This study was carried out on patients undergoing wise-pattern reduction mammoplasty because, among other oncoplastic techniques, it is burdened by a greater risk for complications in the postoperative course [35]. Independently from the skin pattern, any pedicle can be used to achieve a good breast reduction. As reported by Hall-Findlay [36], there are numerous combinations available: the type of pedicle can be different in the case of contralateral symmetrization between two breasts both for various oncoplastic indications and the need for RT in the breast affected by cancer, a factor that affects the stability of the long-term result.

The inclusion criteria were grade II or higher breast ptosis, according to the second Regnault classification [37], excision of breast parenchyma greater than 20% of the breast volume, or an excision of less than 20% of the affected breast with a smaller volume than the contralateral. The exclusion criteria were patient refusal, previous RT, absence of oncological indications for conservative surgery, contraindications to adjuvant RT, connective tissue diseases, and patients with poor compliance with device management.

Clinical, pathological, and surgical data were collected upon informed consent.

The standard schedule of post-operative evaluations included a first admission after three days from hospital discharge and subsequent weekly controls until complete wound healing. The programmed control was antedated upon clinical decision when a clinical evaluation of the operated breast was required.

The number of outpatient clinic admissions and postoperative complications were collected and compared between the two groups.

This study was approved by the Scientific Committee of the Department of Medicine, Surgery and Neurosciences of the University of Siena with a favorable report. Ethical approval was not required because this observational study, using devices already approved for use in daily clinical practice, did not involve any modification of standard treatment protocols.

Enrollment started in January 2022 and ended in January 2023. The patients were divided into two groups, the CD group and the NPWD group, by a simple randomization list.

Patients in the CD group were dressed using sterile medical gauze and adhesive tape.

**Figure 2:**
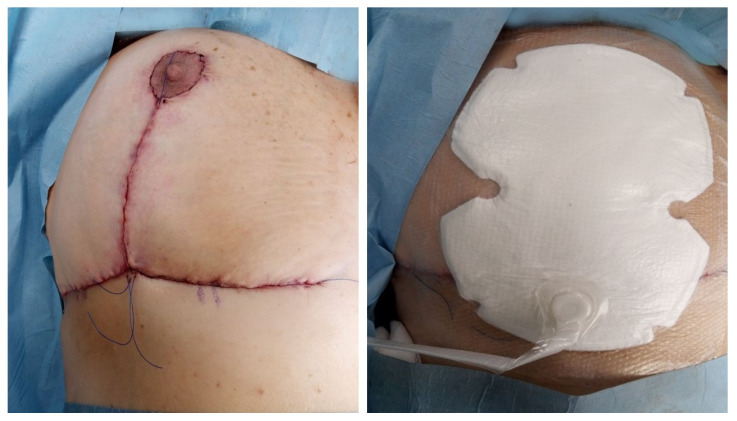
NPWD placement at the end of surgery.

Patients in the NPWD group were treated with a canister-free, single-use negative pressure wound therapy system that delivers −80 mmHg of pressure (Figure 2). After each dressing, all patients wore a surgical bra.

In March 2023, with a minimum follow-up of 2 months, we evaluated all patients’ admissions.

Statistical analysis was performed with SPSS software, Version 27.0 (IBM Corp., Armonk, NY, USA). The simple descriptive statistics include the patients’ socio-demographics, clinical characteristics, and complications. We verified the normal distribution of the continuous variables using the Shapiro–Wilk test; outcome analysis was performed for the continuous variables using Student’s *t*-test and for the discrete variables using the χ^2^ test. *p* values of less than 0.05 were considered statistically significant.

## 3. Results

A total of 100 patients were enrolled, with 52 in the CD group, and 48 in the NPWD group.

The population was homogeneous in the clinic and intra-operative characteristics and the relevant ones are reported in Table 2; no significant differences were found between the groups.

Axillary lymph node surgery was not performed in six patients of the CD group (11.5%) and in three patients of the NPWD group (6.3%) because the tumor was a DCIS with a size < 20 mm, and no investigation of the sentinel lymph node status was required.

Short-term complications occurred in 23 patients: 15 in the CD group (28.8%) and 8 in the NPWD group (16.7%), with no statistical difference between the groups (Table 3). We reported in the CD group five hematomas (9.6%), five seromas (9.6%), four wound dehiscence (7.7%), and 1 Wound Infection (1.9%); in the NPWD group, we reported four hematomas (8.3%), three seromas (6.4%), and one wound dehiscence (2%). We highlighted the statistical differences only between the wound dehiscence rate (*p* = 0.002).

One month after surgery, we evaluated the number of admissions to our ambulatory (Table 4): in the CD group, the patients had made 5.7 ± 1.3 admissions in one month and patients with NPWD had made 3.8 ± 1.1 admissions; this difference was significant (*p* = 0.0009).

## 4. Discussion

The oncological pathway of patients with breast cancer is characterized by a multidisciplinary approach and marked by precise timing to carry out therapies and surgical procedures [38].

The post-surgical course of patients undergoing breast surgery is characterized by multiple admissions to the reference structures for medications and follow-up. This pathway is necessary and conducted within the hospital.

NPWDs, initially designed for the treatment of surgical wounds associated with factors delaying healing, such as diabetes, smoking, and a high BMI (>40), were found to be a valid ally in the management of patients undergoing breast surgery both for treatment and preventive purposes, as reported in a recent meta-analysis by Cagney et al. [33].

In this regard, Tanaydin et al. [39] conducted a randomized study in patients submitted to breast surgery and reported that NPWDs can reduce the overall wound healing complication rate, especially for dehiscence. Moreover, the evidence reported in the literature demonstrates that NPWDs can reduce, as a preventive approach, the incidence of seromas and infections and can promote the healing of breast wounds [40,41]. In a recent randomized study, Pieszko et al. [42] highlighted a significant decrease in surgical-site wound complications within 1 year of surgery and more elastic scar tissue with the preventive application of NPWDs in immediate breast reconstruction.

To the best of our knowledge, few studies have compared NPWDs with standard dressings in oncoplastic breast procedures [40,43,44].

The learning curve of specialists is very important to maximize the benefits of these devices for the correct application of wound dressing and the correct education of patients for the home management of NPWDs. (Video S1)

The study aimed to document whether NPWDs could entail fewer admissions in post-surgical follow-up and guarantee a favorable post-operative course for patients undergoing major oncoplastic surgery, with a lower complication rate.

We noticed how NPWDs in oncoplastic procedures significantly reduced the rate of wound dehiscence, as reported in the existing literature [33,43]. Moreover, although not significantly, it is possible to note a trend of reduction in clinically relevant postoperative total complications in patients treated with NPWDs. Furthermore, our data show that the use of negative pressure dressings significantly reduced the number of admissions to outpatient clinics, a result expected for the functioning and durability of these kinds of dressings. If no complications arise, these devices are replaced every 7 days [32].

Hypothetically higher costs of such devices were not investigated in this study. We are aware that economics could represent a matter of concern, especially compared to CD.

Nevertheless, we believe that further studies are needed to deeply evaluate this aspect from a wider economic perspective, considering not only the raw costs of the devices but also the global advantages derived from the reduction of outpatient clinical admissions and professional care involvement, as well as the faster process of scar healing, and wound stimulation and environmental isolation, leading to lower rates of postoperative complications.

## 5. Conclusions

Our study demonstrates how negative pressure dressings may represent a useful tool in the post-surgical management of oncological patients who undergo breast oncoplastic procedures, ensuring fewer wound dehiscences. In this scenario, the adoption of these dressings, especially in complex patients undergoing high-risk surgery, seems to be increasingly gaining ground, supported by a growing base of literature. Furthermore, the use of these dressings leads to a significant reduction in admissions to post-surgical clinics, promoting a lower use of resources by hospitals and the effective prevention of possible complications.

## Figures and Tables

**Table 1:** Indication and contraindication to conservative oncoplastic surgery.

**INDICATION TO CONSERVATIVE ONCOPLASTIC SURGERY**
Grade II or higher breast ptosis, second Regnault classification
Excision of breast parenchyma greater than 20% of the breast volume
Excision less than 20% but in a breast affected by malignant lesion originally of a smaller volume than the contralateral
**CONTRAINDICATION TO CONSERVATIVE ONCOPLASTIC SURGERY**
Previous RT
Absence of oncological indications for conservative surgery
Contraindications to adjuvant RT
Connective tissue diseases

**Table 2:** Characteristics of the study’s population.

		CD	NPWD
**Patients**		52	48
**Age**		55.8 (26–74)	55.7 (35–76)
**BMI**		24.9 (18.8–32.3)	25.5 (19.4–34.1)
**Comorbidity**			
	*Hypercholesterolemia*	6 (11.5%)	4 (8.3%)
	*Diabetes*	4 (7.7%)	3 (6.3%)
	*Hypertension*	7 (13.5%)	4 (8.3%)
**Smoking**			
	*Never*	35 (67.3%)	37 (77.1%)
	*Ex*	9 (17.3%)	8 (16.7%)
	*Smoker*	8 (15.4%)	3 (6.2%)
**Previous breast surgery**		6 (11.4%)	5 (9.5%)
	*Wide local excision*	3 (5.7%)	2 (4.2%)
	*Contralateral mastectomy*	2 (3.8%)	2 (4.2%)
	*Augmentation*	1 (1.9%)	1 (1.1%)
**Axillary surgery**			
	*Axillary dissection*	8 (15.4%)	8 (16.6%)
	*Sentinel lymph node biopsy*	38 (73.1%)	37 (77.1%)
	*No surgery*	6 (11.5%)	3 (6.3%)

**Table 3:** Short-term complications.

	CD	NPWD	*p*
**Seroma**	5 (9.6%)	3 (6.4%)	0.34
**Dehiscence**	4 (7.7%)	1 (2.0%)	0.002 *
**Hematoma**	5 (9.6%)	4 (8.3%)	0.82
**Wound Infection**	1 (1.9%)	0 (0%)	0.27
**Overall**	15 (28.8%)	8 (16.7%)	0.15

* Statistically significant (*p* < 0.05).

**Table 4:** Ambulatory admissions.

Number of Admissions		
**CD**	**NPWD**	*p*
5.7 ± 1.3	3.8 ± 1.1	0.0009 *

* Statistically significant (*p* < 0.05).

## Data Availability

Not applicable.

## Supplementary Materials

The following supporting information can be downloaded at: https://www.mdpi.com/article/10.3390/medicina59101703/s1, Video S1: NPWD placement at the end of surgery.

## References

[1] Weber W.P., Soysal S.D., El-Tamer M., Sacchini V., Knauer M., Tausch C., Hauser N., Günthert A., Harder Y., Kappos E.A. (2017). First international consensus conference on standardization of oncoplastic breast conserving surgery. Breast Cancer Res. Treat..

[2] Weber W.P., Soysal S.D., Fulco I., Barandun M., Babst D., Kalbermatten D., Schaefer D.J., Oertli D., Kappos E.A., Haug M. (2017). Standardization of oncoplastic breast conserving surgery. Eur. J. Surg. Oncol..

[3] Boughey J.C., Rosenkranz K.M., Ballman K.V., McCall L., Haffty B.G., Cuttino L.W., Kubicky C.D., Le-Petross H.T., Giuliano A.E., Van Zee K.J. (2023). Local Recurrence After Breast-Conserving Therapy in Patients With Multiple Ipsilateral Breast Cancer: Results From ACOSOG Z11102 (Alliance). J. Clin. Oncol..

[4] Kimball C.C., Nichols C.I., Vose J.G., Peled A.W. (2018). Trends in Lumpectomy and Oncoplastic Breast-Conserving Surgery in the US, 2011–2016. Ann. Surg. Oncol..

[5] Casella D., Palumbo P., Sandroni S., Caponi C., Littori F., Capuano F., Grimaldi L., Marcasciano M., Cuomo R. (2022). Positive ROS (Reactive Oxygen Species) Modulator Engineered Device Support Skin Treatment in Locally Advanced Breast Cancer (LABC) Enhancing Patient Quality of Life. J. Clin. Med..

[6] Calabrese C., Casella D., Di Taranto G., Marcasciano M., Kothari A., Sordi S., Barellini L., Torto F.L., Tarallo M., Perra A. (2018). Oncoplastic conservative surgery for breast cancer: Long-term outcomes of our first ten years experience. Eur. Rev. Med. Pharmacol. Sci..

[7] André C., Holsti C., Svenner A., Sackey H., Oikonomou I., Appelgren M., Johansson A.L.V., de Boniface J. (2021). Recurrence and survival after standard versus oncoplastic breast-conserving surgery for breast cancer. BJS Open.

[8] Fitzal F., Bolliger M., Dunkler D., Geroldinger A., Gambone L., Heil J., Riedel F., de Boniface J., Andre C., Matrai Z. (2022). Retrospective, Multicenter Analysis Comparing Conventional with Oncoplastic Breast Conserving Surgery: Oncological and Surgical Outcomes in Women with High-Risk Breast Cancer from the OPBC-01/iTOP2 Study. Ann. Surg. Oncol..

[9] Ritter M., Oberhauser I., Montagna G., Zehnpfennig L., Schaefer K., Ling B.M., Levy J., Soysal S.D., Müller M., López L.C. (2022). Comparison of patient-reported outcomes among different types of oncoplastic breast surgery procedures. J. Plast. Reconstr. Aesthetic Surg..

[10] Mohamedahmed A.Y.Y., Zaman S., Zafar S., Laroiya I., Iqbal J., Tan M.L.H., Shetty G. (2022). Comparison of surgical and oncological outcomes between oncoplastic breast-conserving surgery versus conventional breast-conserving surgery for treatment of breast cancer: A systematic review and meta-analysis of 31 studies. Surg. Oncol..

[11] De La Cruz L., Blankenship S.A., Chatterjee A., Geha R., Nocera N., Czerniecki B.J., Tchou J., Fisher C.S. (2016). Outcomes After Oncoplastic Breast-Conserving Surgery in Breast Cancer Patients: A Systematic Literature Review. Ann. Surg. Oncol..

[12] Nanda A., Hu J., Hodgkinson S., Rainsbury R., Roy P.G. (2020). Oncoplastic breast-conserving surgery for women with primary breast cancer. Cochrane Database Syst. Rev..

[13] Casella D., Fusario D., Cassetti D., Pesce A.L., De Luca A., Guerra M., Cuomo R., Ribuffo D., Neri A., Marcasciano M. (2022). Controlateral Symmetrisation in SRM for Breast Cancer: Now or Then? Immediate versus Delayed Symmetrisation in a Two-Stage Breast Reconstruction. Curr. Oncol..

[14] Hoffmann J., Wallwiener D. (2009). Classifying breast cancer surgery: A novel, complexity-based system for oncological, oncoplastic and reconstructive procedures, and proof of principle by analysis of 1225 operations in 1166 patients. BMC Cancer.

[15] Clough K.B., Kaufman G.J., Nos C., Buccimazza I., Sarfati I.M. (2010). Improving Breast Cancer Surgery: A Classification and Quadrant per Quadrant Atlas for Oncoplastic Surgery. Ann. Surg. Oncol..

[16] Chatterjee A., Gass J., Patel K., Holmes D., Kopkash K., Peiris L., Peled A., Ryan J., El-Tamer M., Reiland J. (2019). A Consensus Definition and Classification System of Oncoplastic Surgery Developed by the American Society of Breast Surgeons. Ann. Surg. Oncol..

[17] Piper M.L., Esserman L.J., Sbitany H., Peled A.W. (2016). Outcomes Following Oncoplastic Reduction Mammoplasty: A Systematic Review. Ann. Plast. Surg..

[18] Panhofer P., Ferenc V., Schütz M., Gleiss A., Dubsky P., Jakesz R., Gnant M., Fitzal F. (2014). Standardization of morbidity assessment in breast cancer surgery using the Clavien Dindo Classification. Int. J. Surg..

[19] Clough K.B., Lewis J.S., Couturaud B., Fitoussi A., Nos C., Falcou M.-C. (2003). Oncoplastic Techniques Allow Extensive Resections for Breast-Conserving Therapy of Breast Carcinomas. Ann. Surg..

[20] Acea-Nebril B., García-Novoa A., Cereijo-Garea C. (2020). Cosmetic sequelae after oncoplastic breast surgery: Long-term results of a prospective study. Breast J..

[21] Haloua M.H., Krekel N.M., Winters H.A., Rietveld D.H., Meijer S., Bloemers F.W., Tol M.P.v.D. (2013). A Systematic Review of Oncoplastic Breast-Conserving Surgery: Current weaknesses and future prospects. Ann. Surg..

[22] Clough K.B., van la Parra R.F.D., Thygesen H.H., Levy E., Russ E., Halabi N.M., Sarfati I., Nos C. (2018). Long-term Results After Oncoplastic Surgery for Breast Cancer: A 10-year Follow-up. Ann. Surg..

[23] Cuomo R., Giardino F.R., Neri A., Nisi G., Brandi C., Zerini I., Jingjian H., Grimaldi L. (2021). Optimization of Prepectoral Breast Reconstruction. Breast Care.

[24] Danino A.M., Coeugniet E. (2008). Letters to the editor: Negative pressure dressing: Some background to a monopole business. Eplasty.

[25] Morykwas M.J., Argenta L.C., Shelton-Brown E.I., McGuirt W.B. (1997). Vacuum-Assisted Closure: A New Method for Wound Control and Treatment: Animal studies and basic foundation. Ann. Plast. Surg..

[26] Apelqvist J., Willy C., Fagerdahl A.-M., Fraccalvieri M., Malmsjö M., Piaggesi A., Probst A., Vowden P. (2017). EWMA Document: Negative Pressure Wound Therapy: Overview, challenges and perspectives. J. Wound Care.

[27] Hopf H.W., Rollins M.D., Robson K., Cripps P., Bardell D., Bhallamudi V.P., Xue R., Purser C.M., Presley K.F., Banasavadi-Siddegowda Y.K. (2007). Wounds: An Overview of the Role of Oxygen. Antioxidants Redox Signal..

[28] Scherer S.S., Pietramaggiori G., Mathews J.C., Orgill D.P. (2009). Short Periodic Applications of the Vacuum-Assisted Closure Device Cause an Extended Tissue Response in the Diabetic Mouse Model. Plast. Reconstr. Surg..

[29] Singh D., Chopra K., Sabino J., Brown E. (2020). Practical Things You Should Know about Wound Healing and Vacuum-Assisted Closure Management. Plast. Reconstr. Surg..

[30] Erba P., Ogawa R., Ackermann M., Adini A., Miele L.F., Dastouri P., Helm D., Mentzer S.J., D’amato R.J., Murphy G.F. (2011). Angiogenesis in Wounds Treated by Microdeformational Wound Therapy. Ann. Surg..

[31] Scalise A., Calamita R., Tartaglione C., Pierangeli M., Bolletta E., Gioacchini M., Gesuita R., Di Benedetto G. (2015). Improving wound healing and preventing surgical site complications of closed surgical incisions: A possible role of Incisional Negative Pressure Wound Therapy. A systematic review of the literature. Int. Wound J..

[32] Galiano R.D., Hudson D.F., Shin J., van der Hulst R., Tanaydin V., Djohan R., Duteille F., Cockwill J.M., Megginson S.M., Huddleston E. (2018). Incisional Negative Pressure Wound Therapy for Prevention of Wound Healing Complications Following Reduction Mammaplasty. Plast. Reconstr. Surg. Glob. Open.

[33] Cagney D., Simmons L., O’leary D.P., Corrigan M., Kelly L., O’sullivan M.J., Liew A., Redmond H.P. (2020). The Efficacy of Prophylactic Negative Pressure Wound Therapy for Closed Incisions in Breast Surgery: A Systematic Review and Meta-Analysis. World J. Surg..

[34] Cuomo R., Grimaldi L., Nisi G., Zerini I., Giardino F.R., Brandi C. (2021). Ultraportable Devices for Negative Pressure Wound Therapy: First Comparative Analysis. J. Investig. Surg..

[35] Toth B.A., Lappert P. (1991). Modified Skin Incisions for Mastectomy: The need for plastic surgical input in preoperative planning. Plast. Reconstr. Surg..

[36] Hall-Findlay E.J. (2002). Pedicles in vertical breast reduction and mastopexy. Clin. Plast. Surg..

[37] Regnault P. (1976). Breast ptosis. Definition and treatment. Clin. Plast. Surg..

[38] Biganzoli L., Cardoso F., Beishon M., Cameron D., Cataliotti L., Coles C.E., Bolton R.C.D., Trill M.D., Erdem S., Fjell M. (2020). The requirements of a specialist breast centre. Breast.

[39] Tanaydin V., Beugels J., Andriessen A., Sawor J.H., van der Hulst R.R.W.J. (2018). Randomized Controlled Study Comparing Disposable Negative-Pressure Wound Therapy with Standard Care in Bilateral Breast Reduction Mammoplasty Evaluating Surgical Site Complications and Scar Quality. Aesthetic Plast. Surg..

[40] Ferrando P.M., Ala A., Bussone R., Bergamasco L., Perinetti F.A., Malan F. (2018). Closed Incision Negative Pressure Therapy in Oncological Breast Surgery: Comparison with Standard Care Dressings. Plast. Reconstr. Surg.-Glob. Open.

[41] Matusiak D., Wichtowski M., Pieszko K., Kobylarek D., Murawa D. (2019). Is negative-pressure wound therapy beneficial in modern-day breast surgery?. Wspolczesna Onkol..

[42] Pieszko K., Pieszko K., Wichtowski M., Cieśla S., Ławnicka A., Jamont R., Boyd J.B., Murawa D. (2023). A Randomized Study Comparing Closed-Incision Negative-Pressure Wound Therapy with Standard Care in Immediate Breast Reconstruction. Plast. Reconstr. Surg..

[43] Wareham C.M., Karamchandani M.M., Ku G.D.L.C., Gaffney K., Sekigami Y., Persing S.M., Homsy C., Nardello S.D., Chatterjee A.M. (2023). Closed Incision Negative Pressure Therapy in Oncoplastic Breast Surgery: A Comparison of Outcomes. Plast. Reconstr. Surg.-Glob. Open.

[44] Gabriel A., Sigalove S.R., Maxwell G.P. (2016). Initial Experience Using Closed Incision Negative Pressure Therapy after Immediate Postmastectomy Breast Reconstruction. Plast. Reconstr. Surg. Glob. Open.

